# Development, Evaluation, and Implementation of a New 3D Printed Tongue Depressor Dispenser

**DOI:** 10.7759/cureus.3764

**Published:** 2018-12-21

**Authors:** Chris Patey, Paul Norman, Nicole Bishop, Michael Bartellas, Adam Dubrowski

**Affiliations:** 1 Emergency Medicine, Carbonear General Hospital, Carbonear , CAN; 2 Emergency Medicine, Carbonear General Hospital, Carbonear, CAN; 3 Medical Education and Simulation, Memorial University of Newfoundland, St. John's, CAN; 4 Otolaryngology Head and Neck Surgery, University of Ottawa, Ottawa, CAN; 5 Emergency Medicine, Memorial University of Newfoundland, St. John's, CAN

**Keywords:** tongue depressor, 3d printing, safety

## Abstract

The purpose of this technical report was two-fold. First was to describe the concept, development and initial implementation of a three-dimensional (3D) printing network focussed on manufacturing simulators and simple devices necessary to the functioning of rural hospital and clinics. Second was to describe the design, fabrication and user-based evaluation of a cost-effective tongue depressor dispenser.

The initial successful setup and implementation of the 3D printing network were modelled using four of the five implementation constructs derived from the Consolidated Framework for Implementation Research (CFIR).

The 3D printing of the tongue depressor dispenser was found to be an effective and economic initiative. Without considering the initial design costs, the materials costs were estimated at $6 Canadian per dispenser. After installation of the dispenser in a busy emergency department, hospital leadership and staff viewed it as a safer option to the current dispense, and more affordable.

## Introduction

This technical report describes the initial development and implementation of a three-dimensional (3D) printing network (MED 3D Network) that focuses on point of care 3D printing manufacturing (PoC3DM) of simulators and clinical devices. Specifically, the report focuses on the development of the first site in the network (Carbonear, Newfoundland and Labrador, Canada) and reviews how the network operates by describing the design and manufacturing of the first collaborative project: tongue depressor dispenser.

The initial site was selected using a set of criteria derived from the Consolidated Framework for Implementation Research (CFIR) [1, 2]. The criteria for selection of the site were based on four of the five CFIR constructs: inner setting, outer setting, characteristics of individuals, and existing processes. Carbonear was an ideal location to initiate the establishment of the MED 3D Network. The Carbonear General Hospital (CGH) is a rural referral center with engaged nurses, progressive physicians and supportive administration who have an existing desire to be involved in innovation. Furthermore, the CGH had an established local rural research unit, referred to as Carbonear Institute for Rural Research and Innovation by the Sea (CIRRIS), that had defined office space and engaged healthcare professionals who were fully supportive of the immediate implementation of the MED 3D Network. CIRRIS is focussed on innovation from front-line driven problems. It was established by the medical director and nursing director at the CGH who are working front-line clinicians. These two highly motivated clinicians were quickly identified as excellent site leads of the CIRRIS 3D design team. The immediate vision for this 3D printing rural site was to address unique product designs that did not exist in the workplace. It was also of interest to explore the possibility of printing unique simulation equipment to provide in-situ training to medical students, residents, and practicing healthcare professionals. With the existing infrastructure and drive, there was an ability to seamlessly integrate and cultivate this technology into the fabric of a rural health care medical facility and community.

Emergency departments (EDs) exist with extensive turnover and thus increased risk of cross-contamination and infectious exposure. One problem identified by the CIRRIS 3D design team was the open exposure of tongue depressors, an essential item in the majority of patient examinations, to cross-contaminate at the emergency examination bedside. A comparable multi-use item, with less capacity for cross-contamination, is the otoscope cover. Within the last decade, and unlike tongue depressors, it now can be clearly found neatly contained in wall units next to otoscope/ophthalmological units. Surprisingly, tongue depressors are still often found in exposed areas of clinical spaces, uncovered in non-sterile, non-cleanable containers with an extreme potential of direct contact and cross contamination. In EDs, with high patient flow, it is essential that clinicians are fully aware of the location of such multi-use items. The tongue depressor collection ideally should be automatic, at hands reach, fully restocked and as sterile as feasibly possible.

The purpose of this technical report is to describe the design, fabrication and user evaluation of a simple tongue depressor dispenser that is easy to clean and reproduce, the use of which reduces the possibility of cross-contamination and create increased order and patient flow in a community ED.

Healthcare-associated infections (HCAI) occur as a result of infection by a number of agents, such as bacteria, parasites, fungi and viruses [3]. The literature states that contamination of environmental surfaces in hospital rooms plays an important role in the transmission of several key HCAIs [4]. Pathogens are capable of surviving on hospital room surfaces and medical equipment for extended periods of time [4]. Routinely, the tongue depressors are kept in jars on the desk or workstation, which increases the likelihood of contamination through healthcare professional hands/gloves. Therefore, the design of the new 3D printed tongue depressor dispenser allows for the release of one tongue depressor at a time with no contamination of additional tongue depressors, potentially leading to a decreased rate of infections. Additionally, the 3D printed tongue depressor dispenser is easy to clean and disinfect leading to the decrease in transmission of HCAIs.

## Technical report

All elements, such as the educational context, inputs, processes, and expected products related to the development and implementation of this model of tongue depressor dispenser, are organized following a modified context, input, process, product (CIPP) model program development and evaluation model [5].

Context

CGH is a rural 80-bed hospital found on the eastern coast of Newfoundland. It is located 00 kilometers from the tertiary referral hospital in the capital city St. John’s. The community of Carbonear has a population of 5000 and the CGH services a catchment population of 40,000. There are four full-time and four part-time emergency physicians, one full-time nurse practitioner, two dedicated paramedics and a maximum of three nurses at one time allocated to the ED.

The 3D printing rural site at the CGH is co-located within CIRRIS and is governed by the same co-directors (medical and nursing). The lab is part of MED 3D Network (https://www.munmed3dnetwork.ca). MED 3D Network was built based on the hub and spokes model, where the spokes are six rural sites and the hub is a central, urban printing laboratory (MUN Med 3D) located in the city of St. John’s, Newfoundland. The urban hub has a multidisciplinary team of engineers, digital designers and researchers that provide structure and ongoing technical support to all six, 3D printing rural sites within the MED 3D Network.

Inputs

The CIRRIS 3D design team, through informal interviews with local ED staff, developed a list of criteria for the proposed dispenser to guide the initial design and prototyping by MUN Med 3D. The requirements were:

● Dispenser must be wall mountable and easily removable,

● Hold standard size tongue depressors,

● Have a visual indicator of how many depressors are currently in the dispenser (“level indicator”),

● Be refillable,

● Be simple to use,

● Be embedded with text indicating use and CIRRIS and MED 3D Network partnership.

Process

Based on the dimensions and requirements conveyed to MUN Med 3D staff via email, a prototype dispenser was modeled using a commercial license of Autodesk Fusion 360 (San Rafael, California). Once the initial design was complete, images of the digital model were sent to the CIRRIS 3D design team for comments and improvements. After several iterative design cycles and confirmation of an acceptable digital model, the model components were converted to stereolithography (.stl) file format and sent electronically to the rural site. At the rural site, the .stl files were sliced using Ultimaker Cura 3 (Geldermalsen, Netherlands), with the resulting G-code loaded on the Ultimaker 3D printer (Geldermalsen, Netherlands) via USB drive for printing. The dispensers were printed using polylactic acid (PLA) filament, with print settings of 0.20 mm layer height at 20% infill, with a 0.4 mm nozzle size.

After the initial model was printed and tested, further modifications were made. These modifications included the increased size of the cutaway for gripping depressors, an increased length of the wall mounts to increase the standoff distance of the dispenser (Video 1).

**Video 1:** Demonstration of the use of the device.

After three months of pilot use of the wall mounted, 3D printed dispenser, the CIRRIS 3D design team conducted two brief product improvement surveys. The surveys were completed by registered nurses (n = 8), primary care paramedics (n = 4), physicians (n =3), and nurse practitioners (n = 1). The first survey (Table 1) explored the functionality of the new dispenser. There were five, five-point Likert scale questions anchored as “Strongly Disagree”, “Disagree”, “Neutral”, “Agree”, and “Strongly Agree”.

**Table 1:** Survey designed to explore the functionality of the new dispenser.

With regards to the 3D Printed Wall-Mounted Tongue Depressor Dispenser	Strongly Disagree	Disagree	Neutral	Agree	Strongly Agree
It functions effectively in the ED?					
It is mounted in a convenient and appropriate location?					
The design and location reduces the risk of contamination during use?					
It is technically easier to clean and can be done so in a more timely manner than the previously mounted dispenser?					
The tongue depressors are easily removed from the dispenser?					

**Figure 1:**
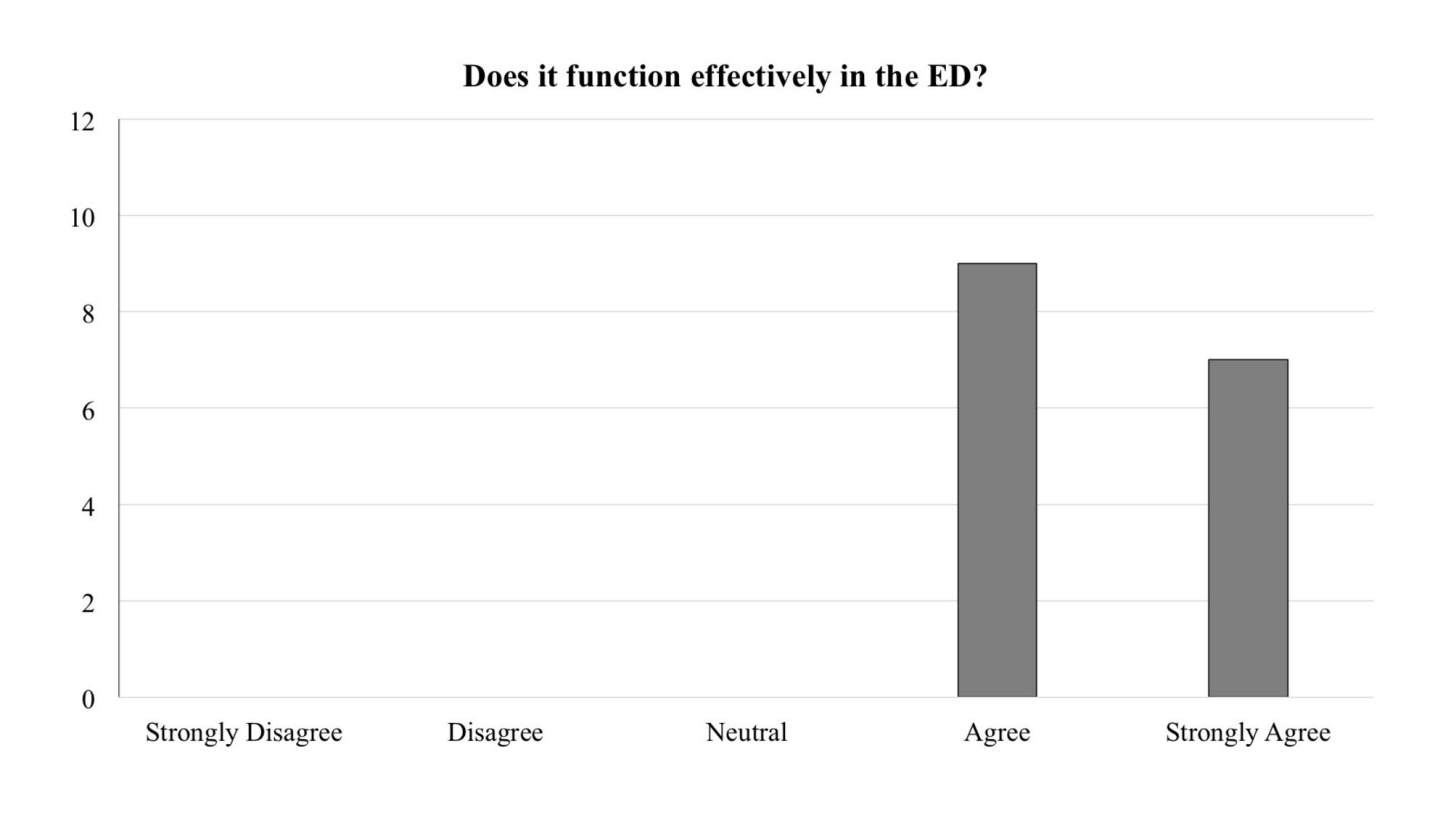
Frequency distribution of answers for question one of the survey designed to explore the functionality of the new dispenser (Total responders n = 16). The question was prefaced with the following: “With regards to the 3D printed wall-mounted tongue depressor dispenser…”

**Figure 2:**
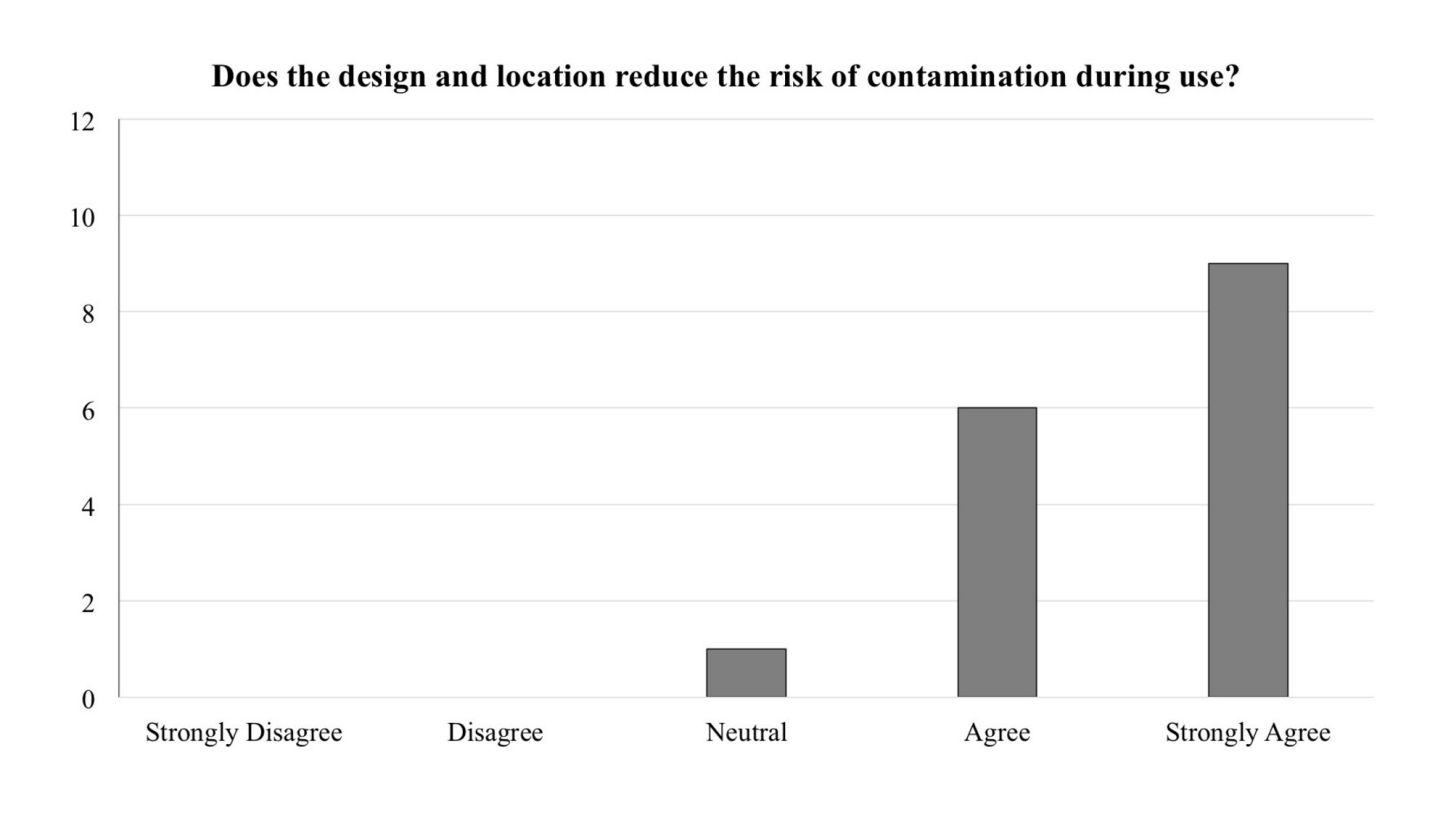
Frequency distribution of answers for question two of the survey designed to explore the functionality of the new dispenser (Total responders n = 16). The question was prefaced with the following: “With regards to the 3D printed wall-mounted tongue depressor dispenser…”

**Figure 3:**
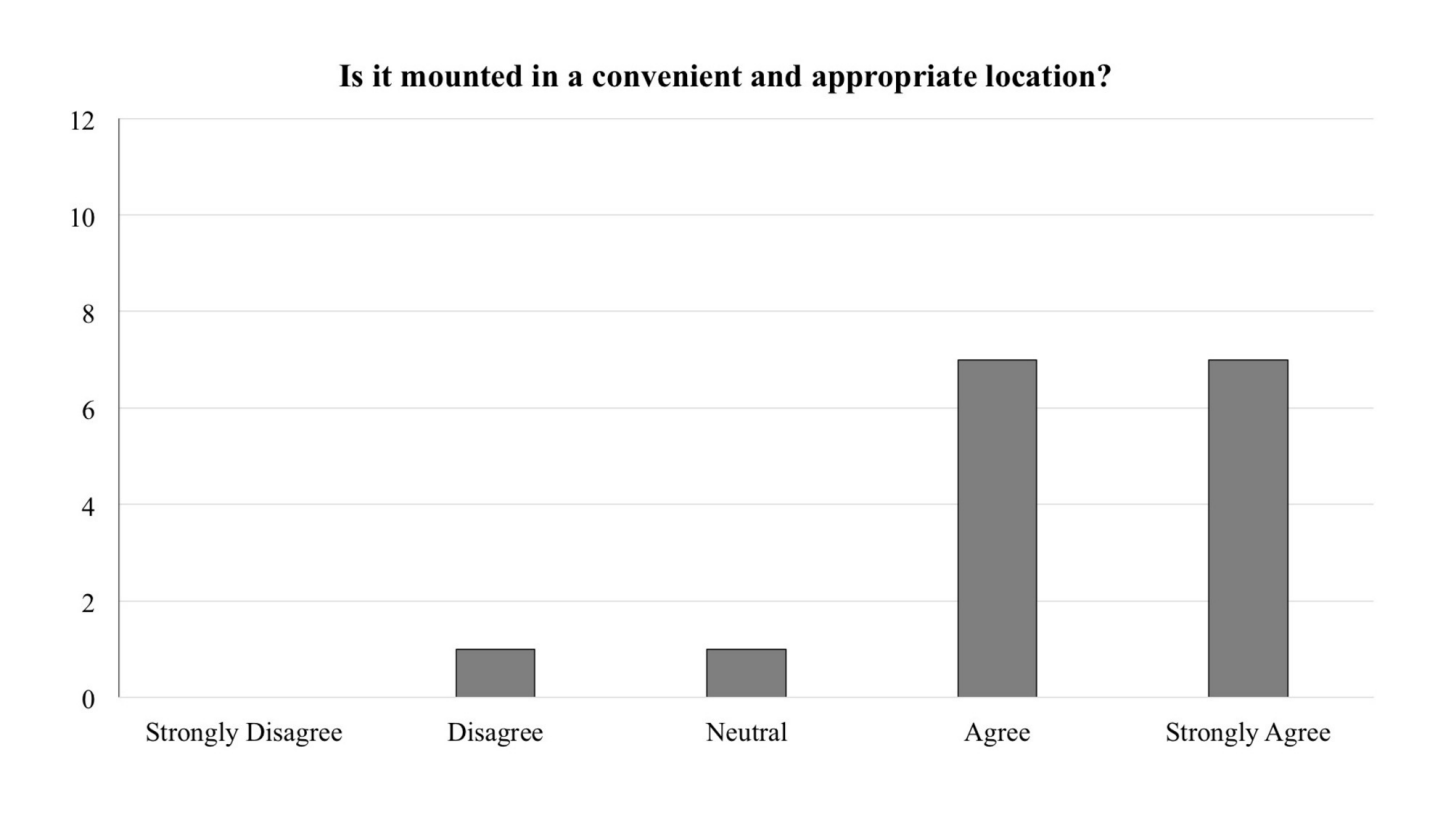
Frequency distribution of answers for question three of the survey designed to explore the functionality of the new dispenser (Total responders n = 16). The question was prefaced with the following: “With regards to the 3D printed wall-mounted tongue depressor dispenser…”

**Figure 4:**
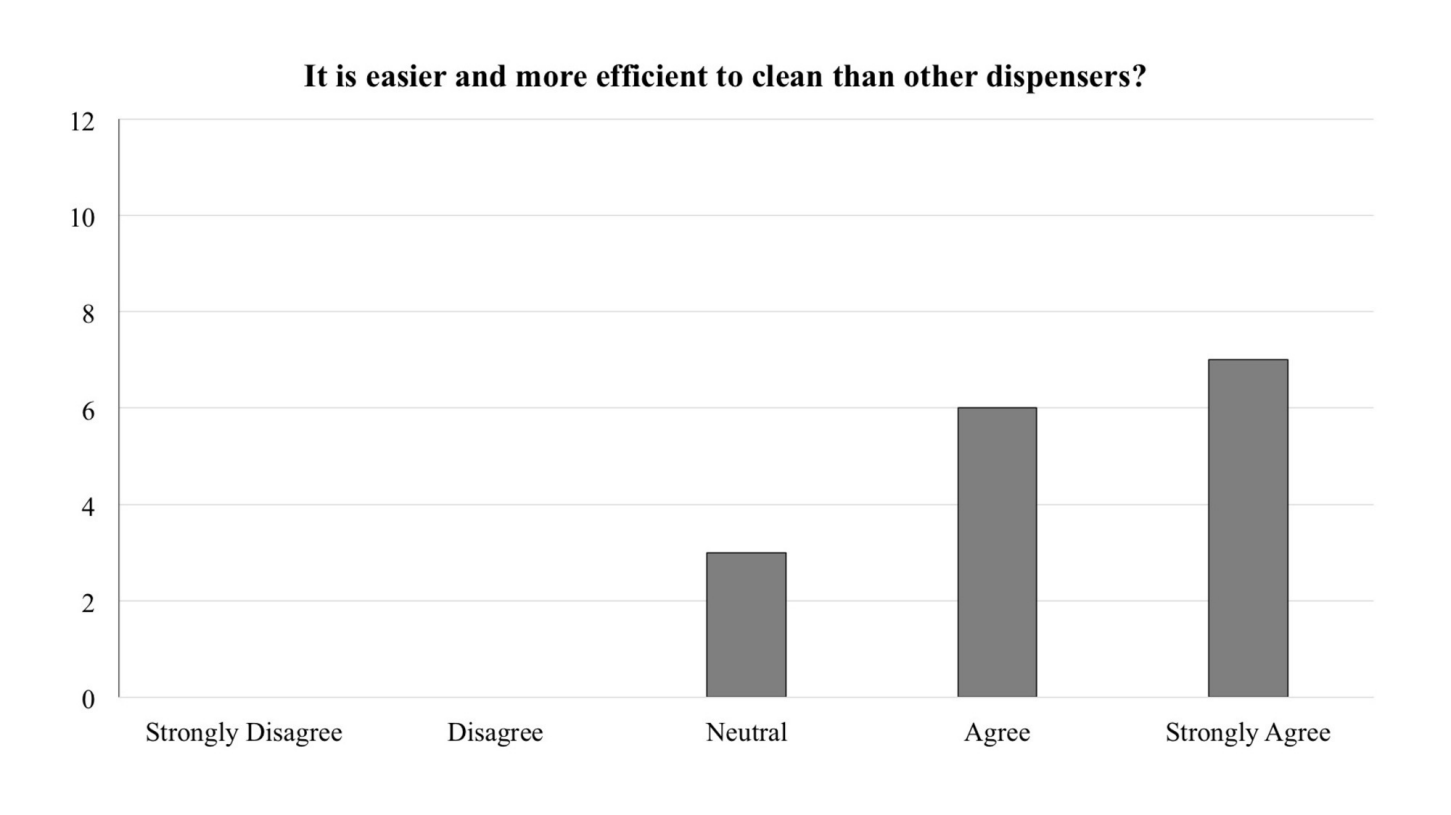
Frequency distribution of answers for question four of the survey designed to explore the functionality of the new dispenser (Total responders n = 16). The question was prefaced with the following: “With regards to the 3D printed wall-mounted tongue depressor dispenser…”

**Figure 5:**
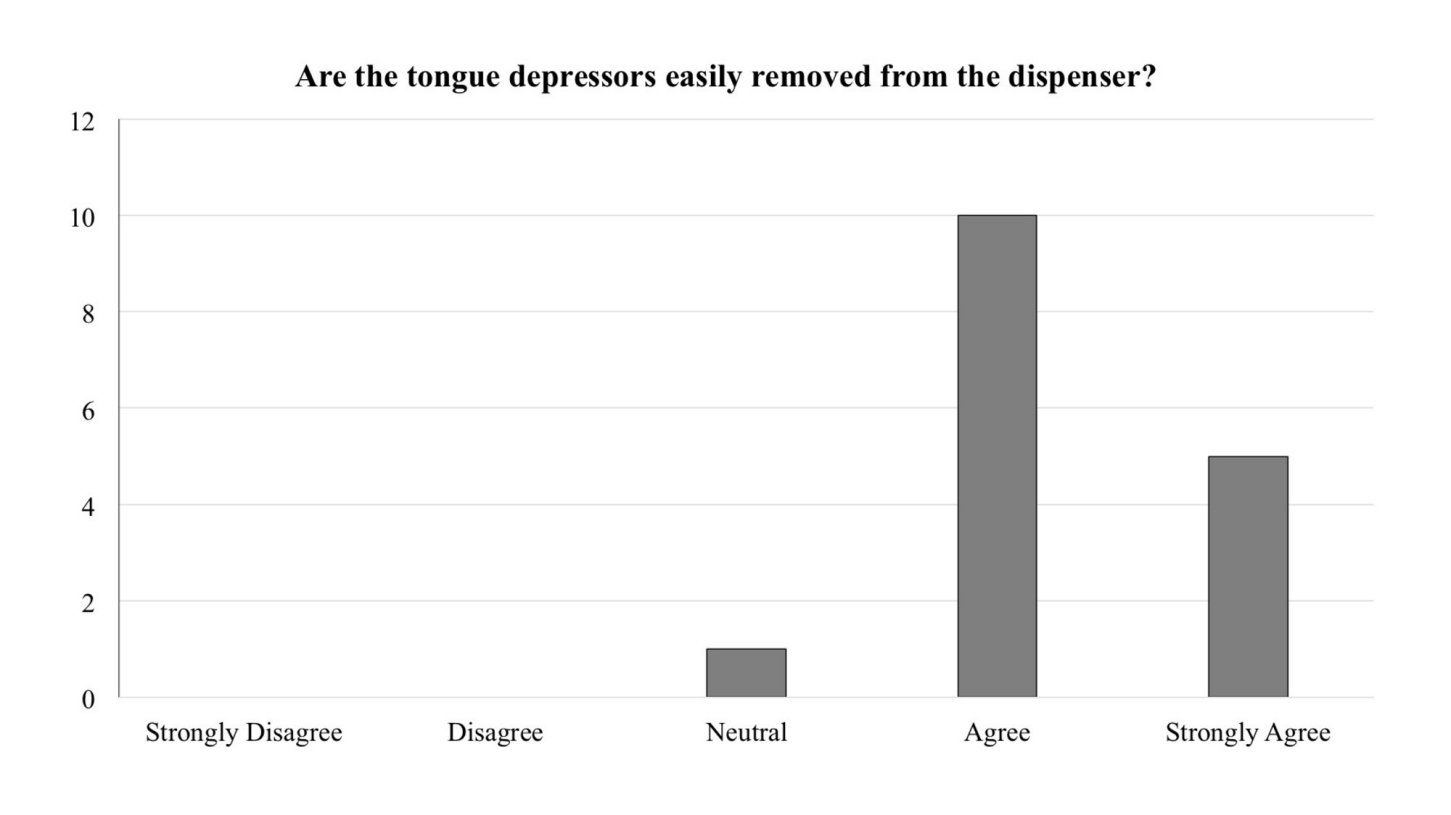
Frequency distribution of answers for question five of the survey designed to explore the functionality of the new dispenser (Total responders n = 16). The question was prefaced with the following: “With regards to the 3D printed wall-mounted tongue depressor dispenser…”

The frequency of responses for each question is reported in Figures 1-5. In general, across all five questions, the responders reported agreement or strong agreement with the statements. In addition, based on the survey, we have noted two areas for improvement. Specifically, one person disagreed with the statement that the mounting location was appropriate (Figure 3), and one person disagreed with the statement that the depressors are easily removed from the dispenser (Figure 5).

The purpose of the second survey was to compare the practices of using the new dispenser and compare them to the current practices. It was a five-question, dichotomous (yes/no) survey. The survey and the results are illustrated in Table 2. The results revealed that the 3D printed dispensers are used by the staff, in general, neither the old nor the new dispensers are routinely cleaned, but it is perceived that the 3D printed dispenser is less contaminated than the practices that it is designed to replace. In summary, 95% of the responders agreed that the 3D printed tongue depressor dispenser should be used within their clinical setting.

**Table 2:** Results of the survey designed to compare the practices of using the new dispenser and compare them to the current practices (Total responders n = 16).

Have you ever or do you currently?	Yes	No
Refill the new 3D printed tongue depressor dispenser?	12	4
Clean the new 3D printed tongue depressor dispenser?	1	15
Clean the old tongue depressors containers?	2	14
Think tongue depressors are less contaminated with the new dispenser?	15	1
Recommend continuing the use of the new 3D printed tongue depressor dispensers?	15	1

Product

For the current version of the dispenser, a total 130 g of PLA was used at an approximate cost of $3.25 (all prices in Canadian dollars), and 30 g of polyvinyl alcohol (PVA) was used at an approximate cost of $2.68 (at $0.03/gram for PLA and $0.12/gram for PVA). The print took 16 hours and 11 minutes to complete at a total material cost of $5.93. With subsequent in-clinic use, some improvements have been identified for this version including changing the depressor access cut-out from the left to the right side, improving cap fit, and reducing overall material usage.

The CIRRIS 3D design team demonstrated the working tongue depressor dispenser to ED management staff who agreed to install and test the functionality in the assessment rooms of the ED. There are currently 11 tongue depressor dispensers in use in the hospital’s ED. CIRRIS 3D design team also reached out to a collaborating physician in a nearby medical clinic and has installed one dispenser in an assessment space at that clinic.

## Discussion

The purpose of this technical report was two-fold. First was to describe the concept, development and initial implementation of a 3D printing network focussed on manufacturing simulators and simple devices necessary to the functioning of rural hospital and clinics. Second was to describe the design, fabrication and user-based evaluation of a cost-effective tongue depressor dispenser.

Through the implementation of the first six rural sites that constitute the MED 3D Network, the network was able to develop an implementation strategy based on indicators for success. We followed four of the five implementation constructs, as described by CFIR [1,2], to draft criteria for successful implementation. Although this technical report describes the development of a single device that was selected as a first project, since April 2018, the CIRRIS 3D design team, in collaboration with MUN Med 3D designers and engineers have completed seven similar projects.

The 3D printing of the tongue depressor dispenser was found to be an effective and economic initiative. Without considering the initial design costs, the materials costs were estimated at $6 per dispenser. This is drastically less than the commercially available units at $200 Canadian [6]. This low-cost solution was viewed by the hospital leadership and staff as not only less expensive but safer, as they were built based on the needs of the users. This need-driven manufacturing approach also facilitates further saving for the hospital because of the reduced need to stock the dispensers as they can be produced on demand. Finally, as more clinics near the CIRRS site, as well as within the MED 3D Network, start using the device, the designs can be easily improved to better serve local, point of care needs.

However, the impact of this initiative is much larger than the point of care 3D printing. Often rural centers have difficulty bridging the gap and establishing a connection to academic centers. The requirement to cover the extensive healthcare burden often outweighs any academic interests. The development and implementation of a rural 3D printing network, similar to the one described in this report, may serve as a realization that rural centers can also contribute to academic research and be at the forefront of technology. In fact, this network shows the future potential for point of care 3D printing in rural and remote areas of healthcare delivery. Local problems and needs are identified, solutions are co-developed with a bioengineering team in an urban lab, printing takes place in the rural site with immediate evaluation and feedback, and refinements can be made on-site or at the urban site. The existence of such 3D printing lab, embedded in a support network, seamlessly incorporated into a rural research unit such as CIRRIS; it also directly inspires clinicians and all members of healthcare to become engaged in research and to improve local and global community health.

## Conclusions

This technical report described the initial implementation of a unique network of rural 3D printing sites and a central biomedical engineering 3D printing hub. We argue that in a rural and remote context, this model facilitates need-based innovation, productivity, and improved designs of simulators and clinical devices.

The co-development of the tongue depressor dispenser was used to illustrate such successful collaboration within the network. The dispenser was evaluated as a functionally superior device when compared to commercially available devices, as it was designed and built to address their shortcomings. The device was manufactured at a fraction of the cost and can be modified as more users are using it in different contexts.
